# Parsonage‐Turner‐syndrome associated with *Mycoplasma pneumoniae* infection

**DOI:** 10.1002/rcr2.70071

**Published:** 2024-12-01

**Authors:** Fabian Leo, Christian Grohé

**Affiliations:** ^1^ Klinik für Pneumologie Evangelische Lungenklinik Berlin Berlin Germany

**Keywords:** brachial plexus neuritis, community‐acquired pneumonia, mycoplasma, neuralgic amyotrophy

## Abstract

Neurological complications in the course of community‐acquired pneumonia indicate that *Mycoplasma pneumoniae* may be the causative pathogen. Parsonage‐Turner‐Syndrome, characterized by neuralgic shoulder pain and amyotrophy, has rarely been reported in this context.

## Graphical abstract

We present a case of Parsonage‐Turner‐Syndrome (PTS) as a rare neurological complication of pneumonia due to *Mycoplasma pneumonia*.

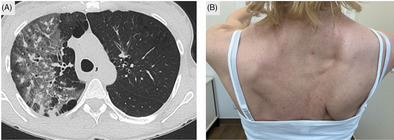

## CLINICAL IMAGE

**FIGURE 1:**
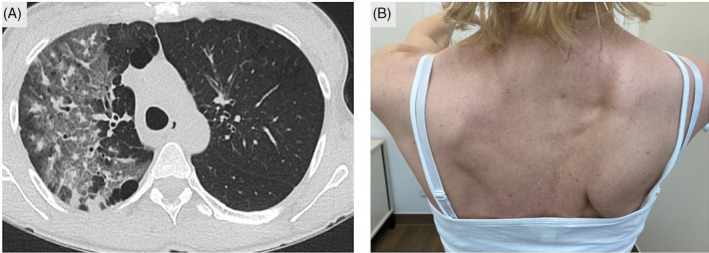
(A) Computed tomography showing extensive ground glass opacities and small, patchy consolidations in the upper lobe of the right lung. (B) Right‐sided amyotrophy of dorsal shoulder muscles and winged scapula (scapula alata).

A 45‐year‐old female patient presented with a 10‐day history of fever and cough that had persisted despite treatment with an oral cephalosporin. Computed tomography imaging was consistent with pneumonia (Figure 1A) and multiplex‐PCR of the bronchoalveolar lavage fluid yielded a positive result for *Mycoplasma pneumoniae* DNA. The administration of levofloxacin resulted in clinical improvement within a few days. During hospitalization, the patient experienced acute pain in her right shoulder, which radiated to her upper arm. Two weeks later, she reported pain relief but muscle weakness upon arm elevation. Physical examination was notable for amyotrophy of the right shoulder with scapular winging (Figure 1B). Serology revealed elevated antibodies against *M. pneumoniae* (IgG 70.70 U/mL [reference <20 U/mL], IgA 22.90 U/mL [<10 U/mL], IgM > 150.00 U/mL [< 13 U/mL]). Given the distinctive clinical presentation and findings, a diagnosis of parainfectious neuralgic amyotrophy, also known as Parsonage‐Turner syndrome (PTS), was established. PTS is an immune‐mediated inflammatory disorder of the brachial plexus.^1^ It represents a rare manifestation within the broad spectrum of neurological diseases associated with *M. pneumoniae* infection.^2^ At the last follow‐up with the patient, 6 months after the initial presentation, she reported moderate functional improvement, requiring continued physical therapy.

## AUTHOR CONTRIBUTIONS

Fabian Leo prepared the figures and wrote the initial manuscript draft. Fabian Leo and Christian Grohé critically revised the manuscript. Fabian Leo and Christian Grohé reviewed and approved the final version.

## CONFLICT OF INTEREST STATEMENT

None declared.

## ETHICS STATEMENT

The authors declare that appropriate written informed consent was obtained for the publication of this manuscript and accompanying images.

## Data Availability

Research data are not shared.
